# The clinical use of the platelet/lymphocyte ratio and lymphocyte/monocyte ratio as prognostic predictors in colorectal cancer: a meta-analysis

**DOI:** 10.18632/oncotarget.15311

**Published:** 2017-02-14

**Authors:** Ya-Huan Guo, Hai-Feng Sun, Yan-Bing Zhang, Zi-Jun Liao, Lei Zhao, Jie Cui, Tao Wu, Jian-Rong Lu, Ke-Jun Nan, Shu-Hong Wang

**Affiliations:** ^1^ Department of Medical Oncology, The First Affiliated Hospital of Xi’an Jiaotong University, Xi’an, Shaanxi, 710061, P.R. China; ^2^ First Department of Medical Oncology, Shaanxi Provincial Tumor Hospital, Xi’an, 710061, P.R. China; ^3^ Third Department of Medical Oncology, Shaanxi Provincial Tumor Hospital, Xi’an, 710061, P.R. China; ^4^ Department of Molecular Physiology and Biophysics, Holden Comprehensive Cancer Center, University of Iowa Carver College of Medicine, Iowa City, IA, USA; ^5^ Department of Oncology, Yan’an University Affiliated Hospital, Yan’an, 716000, P.R. China; ^6^ Third Department of Pathology, Shaanxi Provincial Tumor Hospital, Xi’an, 710061, P.R. China

**Keywords:** platelet/lymphocyte ratio, lymphocyte/monocyte ratio, colorectal cancer, prognostic predictor, inflammatory markers

## Abstract

**Background:**

Conflicting evidence exists regarding the effects of platelet/lymphocyte ratio (PLR) and lymphocyte/monocyte ratio(LMR) on the prognosis of colorectal cancer (CRC) patients. This study aimed to evaluate the roles of the PLR and LMR in predicting the prognosis of CRC patients via meta-analysis.

**Methods:**

Eligible studies were retrieved from the PubMed, Embase,andChina National Knowledge Infrastructure (CNKI) databases, supplemented by a manual search of references from retrieved articles. Pooled hazard ratios (HR) with 95% confidence intervals (95% CI) were calculated using the generic inverse variance and random-effect model to evaluate the association of PLR and LMR with prognostic variables in CRC, including overall survival (OS), cancer-specific survival (CSS) and disease-free survival (DFS).

**Results:**

Thirty-three studies containing 15,404 patients met criteria for inclusion. Pooled analysis suggested that elevated PLR was associated with poorer OS (pooled HR = 1.57, 95% CI: 1.41 – 1.75, *p*< 0.00001, I^2^=26%) and DFS (pooled HR = 1.58, 95% CI: 1.31 – 1.92, *p*< 0.00001, I^2^=66%). Conversely, high LMR correlated with more favorable OS (pooled HR = 0.59, 95% CI: 0.50 – 0.68, *p*< 0.00001, I^2^=44%), CSS (pooled HR = 0.54, 95% CI: 0.40 – 0.72, *p*< 0.00001, I^2^=11%) and DFS (pooled HR = 0.82, 95% CI: 0.71– 0.94,*p*=0.005, I^2^=29%).

**Conclusions:**

Elevated PLR was associated with poor prognosis, while high LMR correlated with more favorable outcomes in CRC patients. Pretreatment PLR and LMR could serve as prognostic predictors in CRC patients.

## INTRODUCTION

CRC represents the third most common cause of cancer-related death in men and women in the united states [1]. It is estimated that 134,490 new cases will be diagnosed and 49,190 deaths will occur in 2016 [1]. Despite advances in surveillance, diagnosis and treatment of CRC, a large number of the patients are still diagnosed at an advanced stage and thus the therapeutic options are limited, resulting in a 5-year survival rate of only about 65% much lower than expected [2]. The discovery of prognostic factors is of clinical importance to guide therapeutic options and surveillance strategies. However, the prognoses of CRC patients with similar clinicopathologic characteristics vary widely due to high heterogeneity in tumor biology [3]. Currently, the discovery of prognostic biomarkers mainly depends on surgical specimens, which may not be representative of the veritable burden of CRC [4]. In addition, as many prognostic factors are evaluated postoperatively, there are still pending circulating biomarkers of early predicting clinical outcome.

Recently, there has been intense interest in the prognostic value of peripheral blood biomarkers in CRC. Inflammation has been reported to be involved in carcinogenesis and disease progression [5] and local cancer-related inflammation can be reflected by a systemic inflammatory response (SIR). Nearly a third of cancer patients have thrombocytosis at diagnosis and aberrant activation of platelets has been shown to be associated with CRC [6, 7]. Lymphocytes are essential components of the tumor microenvironment, which contributes to carcinogenesis [8]. Monocytes have been reported to influence CRC progression and can be used to predict prognosis [9, 10]. PLR and LMR, two representative indices of SIR, have been found to impact survival in a variety of solid malignancies [11–14], including CRC [15, 16]. As the collection of circulating inflammatory markers, including PLR and LMR, is simple, noninvasive, and easily accessible. Circulating levels of inflammatory markers have been investigated as applicable and cost-effective prognostic predictors in cancer patients [17]. Although the underlying mechanisms of altered PLR and LMR in CRC development remains unknown, numerous studies have investigated their value as prognostic factors and markers for predicting response to therapy. However, the results of these studies are conflicting [16, 18, 19]. Therefore, a comprehensive evaluation of the literature is warranted.

In the present study, this meta-analysis represents the most comprehensive and up-to-date review on the prognostic value of PLR and LMR in CRC. The results of this study showed that elevated PLR and LMR were associated with poor and favorable prognosis in CRC, respectively, suggesting that these two factors might be used as prognostic factors in CRC patients and applied in surveillance programs.

## RESULTS

### Search results

**Figure 1:**
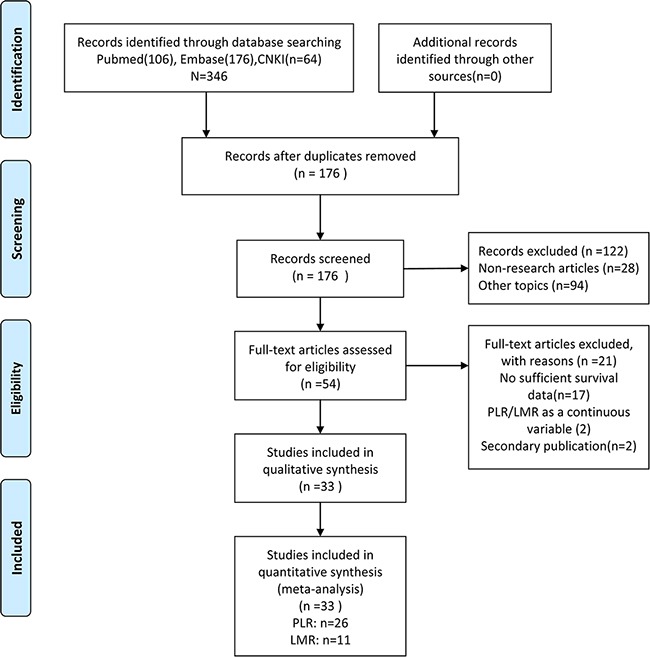
Flow- diagram shows the selection of literature for meta-analysis

Cohen's kappa for inter-reviewer agreement was 0.80 (95% CI=0.69 to 0.93). The literature search process is summarized in a PRISMA flow diagram (Figure 1). Initial assessment of titles and abstracts identified 346 potentially relevant publications which included 170 duplicates, 94 irrelevant studies, and 28 non-research articles. After further screening of full-texts of the remaining 54 articles, 21 papers were excluded due to insufficient survival data or for being a secondary publication. Altogether, 33 studies [3, 16, 18–48] were finally selected for inclusion. Among these studies, 22 investigated PLR, 8 studied LMR and 3 evaluated both PLR and LMR.

### Study description

The basic features of the 33 studies are summarized in Table 1. In total, 15,404 patients were included. All included studies were retrospective cohorts. Among these studies, 2 were published in 2012, 4 in 2013, and the remaining 27 (82%) were published in 2014 or later. Sample sizes ranged from 57 to 5336 patients. The mean or median age of subjects ranged from 49 to 71.3 years. The mean or median follow-up duration ranged from 10.4 to 68 months. Patients in 23 studies [3, 16, 23, 24, 26, 27, 29–37, 40–44, 46–48] were Asian, while subjects were Caucasian in the other 10 studies [18–22, 25, 28, 38, 39, 45]. 6 studies [16, 41–44] included all CRC stages; 16 studies [3, 18, 20, 21, 23, 24, 27, 28, 30, 31, 33, 34, 37, 40, 45, 48] only included non-metastatic CRC; 10 studies [19, 22, 26, 29, 35, 36, 38, 39, 45–47] only included metastatic CRC; and 1 study [45] included two cohorts evaluating the outcomes of both non-metastatic and metastatic CRC. Twenty three studies analyzed PLR as a single dichotomous cut-off (group 1), while three studies [3, 38, 48] defining three risk categories with two cut-offs reported a single HR of PLR (group 2). All studies evaluated LMR as a dichotomous cut-off.

**Table 1:** Baseline characteristics of studies included in this meta-analysis

Study Published year	Country Duration	Sample size Median age	Main treatment Tumor site	Study design Clinical stage	Outcome indices Survival analysis	Follow-up (median and range)	Cut-off value	determine the cut-off value	inflammatory disorders	Study quality#
Baranyai *et al*. 2013	USA 2001-2011	336 CRC:67	CSR CRC	Retrospective N	OS,DFS MVA	67	PLR:300	RPS	No	6
Baranyai *et al*. 1 2013	USA 2001-2011	118 mCRC:61*	mCRC	Retrospective M	OS MVA	NR	PLR:300	RPS	No	6
Carruthers *et al*. 2012	UK 2000-2005	115 63.8(32.3–81.1)*	NeoCRT/adjCT +CSR RC	Retrospective N	OS,DFS UVA	37.1	PLR:160	RPS	NR	6
Chan et al. 2016	Australia 1998-2012	1623 NR	CRT +CSR CRC	Retrospective N	OS PLR:UVA;LMR:MUV	52(27-92)	PLR:258 LMR:2.38	MaxStat analysis	NR	7
Choi et al. 2015	Canada 2004-2012	549 68.7(68.3-98.6)	CSR CRC	Retrospective N	OS,RFS/DFS UVA	48(0-124.8)	PLR:295	MaxStat analysis	NR	8
Chen et al. 2015	China 2010-2014	205 NR	CSR CRC	Retrospective N	RFS/DFS MVA	NR	PLR:176	ROC analysis	NR	6
Cui et al. 2015	China 2007-2011	822 NR	CSR±adjCT/CRT CRC	Retrospective N	OS,RFS/DFS MVA	NR	PLR:194	ROC analysis	NO	7
Duan et al. 2014	China 2007-2008	57 71.3*	CSR CRC	Retrospective NM	OS MVA	NR	PLR:250	NR	NR	5
Kwon et al. 2012	South Korea 2005-2008	200 64(26–83)	CSR±adjCT/CRT CRC	Retrospective N	OS MVA	33.6	PLR:<150 / 150-300 / >300	NR	NR	8
Li et al. 2016	China 2007-2014	5,336 59(51–66)	CSR±adjCT CRC	Retrospective N	OS,DFS MVA	55.2	PLR:219 LMR:2.83	X-tile software	NO	9
Li et al. 2015	China 2003-2012	110 62.9*	PSR+CT CC	Retrospective M	OS MVA	10.4(0.9-122.2)	PLR:162	NR	NR	7
Lin et al. 2016	China 2005-2013	488 54(37-72)	CT CRC	Retrospective M	OS MVA	23.5(4.3–32.8)	LMR:3.11	ROC	NO	9
Liu et al. 2013	China 2005-2011	140 54.1*	CSR CRC	Retrospective NM	OS MVA	NR	PLR:250	NR	NR	6
Luo et al. 2014	China 2006-2010	162 NR	NR CRC	Retrospective NM	OS MVA	NR	PLR:250	NR	NR	5
Mori et al. 2015	Japan 2007-2011	157 67(35-89)	CSR CRC	Retrospective N	DFS UVA	20.5(0.2–62.4)	PLR:150	RPS	NO	7
Neal et al. 2015	UK 2006-2010	302 64.8*(26-85)	CSR±CT CRLM	Retrospective M	OS,CSS UVA	29.7(4-96)	PLR:<150 / 150-300 / >300 LMR:2.35	PLR:RPC LMR:ROC	NO	8
Neofytou *et al*. 2014	UK 2005-2012	140 NR	NeoCT/adjCT +CSR CRLM	Retrospective M	OS,DFS MVA	33(1-103)	PLR:150	ROC analysis	NO	9
Neofytou *et al*. 2015	UK 2005-2012	140 NR	NeoCT/adjCT +CSR CRLM	Retrospective M	OS,CSS MVA DFS UVA	33(1-103)	LMR:3	ROC analysis	NO	9
Ni et al. 2016	China 2010-2015	148 60.2*(20-74)	CT CRC	Retrospective M	OS MVA	12(0.2-67)	PLR:174	RPS	NO	8
Ozawa *et al*. 2015	Japan 2000-2010	234 NR	CSR CRC	Retrospective N	DFS,CSS MVA	64(1-173)	PLR:25.4	ROC analysis	NO	9
Ozawa *et al*. 1 2015	Japan 1997-2012	117 NR	CSR CRC	Retrospective M	DFS,CSS MVA	39(4-170)	LMR:3	ROC analysis	NO	9
Passardi *et al*. 2016	Italy NR	289 NR	CT CRC	Prospective M	OS,PFS MVA	NR	PLR:169	X-tile software	NR	8
Shibutani *et al*. 2015	Japan 2005-2010	104 64(27-86)	CT CRC	Retrospective M	OS MVA	22.4(2.6-69.5)	LMR:3.38	ROC analysis	NR	6
Son et al. 2013	South Korea 2005-2007	624 NR	CSR CRC	Retrospective N	OS,DFS MVA	42(1-66)	PLR:300	NR	NR	7
Song et al. 2015	South Korea 2006-2003	177 52(25-81)	RVS CRC	Retrospective M	OS UVA	3.1(0.1-33.3)	LMR:3.4	ROC analysis	NR	7
Stotz *et al*. 2014	Austria 1996-2011	372 64(27-95)	CSR CR	Retrospective N	OS MVA	68(1-190)	LMR:2.14	ROC analysis	NR	8
Sun et al. 2014	China 2005-2008	255 59.47*	CSR CC	Retrospective N	OS,DFS MVA	NR	PLR:<150 / 150-300 / >300	NR	NR	7
Szkandera *et al*. 2014	Austria 1996-2011	372 64(27-95)	CSR CC	Retrospective N	OS MVA	68(1-190)	PLR:225	ROC analysis	NR	8
Toiyama *et al*. 2013	Japan 2001-2012	84 64.5(33-80)	CRT+CSR RC	Retrospective N	OS,DFS UVA	56(2-147)	PLR:150	RPS	NR	7
Xiao et al. 2015	China 2004-2011	280 NR	CSR RC	Retrospective N	DFS MVA	52(0.5-106.37)	LMR:3.78	median value	NR	7
Ying et al. 2014	China 2005-2010	205 NR	CSR CRC	Retrospective N	RFS,OS,CSS MVA	NR	PLR:176	ROC analysis	NO	7
You et al. 2016	China 2005-2011	1314 66*	CSR CRC	Retrospective NM	DFS,OS MVA	56.9	PLR:150	RPS	No	8
Yu et al. 2016	China 2011-2014	125 49(18-72)	CT CRC	Retrospective M	PFS,OS MVA	NR	LMR:3.6	ROC analysis	NO	6
Zou et al. 2016	China 2006-2012	216 NR	CSR CRC	Retrospective NM	OS MVA	38(3′85 )	PLR: 246.36	ROC analysis	No	8

Notes: Tumor site : CRC colorectal cancer, mCRC metastatic colorectal cancer, CC colon cancer, RC rectal cancer, CRLM colorectal liver metastases. Treatment: CSR curative surgical resection, PSR palliative surgical resection, CRT chemoradiotherapy, CT chemotherapy, neoCRT neoadjuvant chemoradiotherapy, adjCT adjuvant chemotherapy, RVS Rhus verniciflua stokes. Study design: prospective, retrospective Clinical stage: N nonmetastatic, M metastatic, NM nonmetastatic and metastatic.Outcome indices: OS overall survival, DFS disease-free survival, CSS cancer specific survival, PFS progression-free survival, RFS recurrence-free survival.Survival analysis: MVA multivariate analysis, UVA univariate analysis. Determine the cut-off value: RPS refer to the previous study, NR not reported, ROC receiver operating curve analysis, X-tile 3.6.1 software R package MaxStat #Study quality was determined based on the Newcastle-Ottawa Scale (range, 1–9) *Mean

### Impact of PLR on OS and DFS in CRC Patients

**Figure 2:**
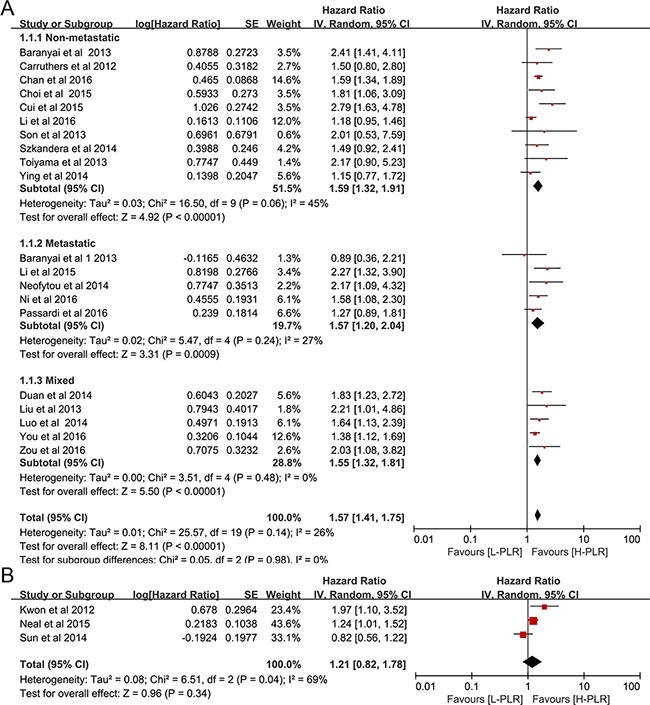
Forest plot reflects the association between PLR and OS **A**. group 1, a single cutoff for PLR. **B**. group 2, two cutoffs for PLR.

**Figure 3:**
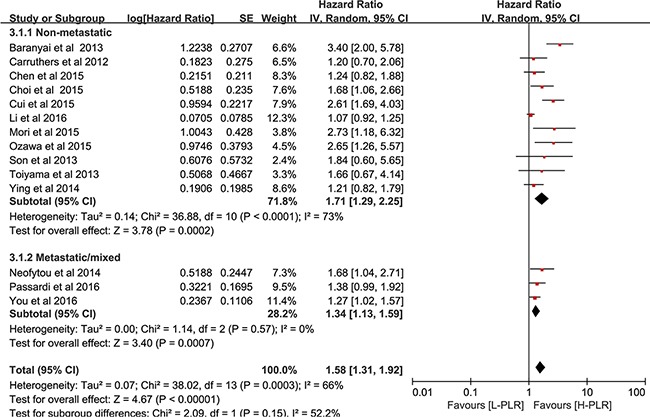
Forest plot reflects the association between PLR and DFS

Twenty studies [16, 18–21, 23, 24, 28, 29, 31, 32, 37, 39–45] in group1, which included 12,760 CRC patients, reported an association between PLR and OS. As seen in Figure 2, the analysis of pooled data showed that elevated PLR was correlated with poor OS in group1 (pooled HR = 1.57, 95% CI: 1.41-1.75, *p*< 0.00001, I^2^=26%, Figure 2A). Furthermore, the results of subgroup indicated that increased PLR was a marker for poor prognosis in non-metastatic CRC (pooled HR = 1.59, 95% CI: 1.32 – 1.91, *p*< 0.00001, Figure 2A), metastatic CRC (pooled HR = 1.57, 95% CI: 1.20 – 2.04, *p*< 0.00001, Figure 2A) and patients at all stages (pooled HR = 1.55, 95% CI: 1.32 – 1.81, *p*< 0.00001, Figure 2A). For studies in group 2 (two cut-offs, usually <150, 150–300, >300), the pooled HR for OS per risk category was 1.21 (95% CI, 0.82–1.78, *p* = 0.10, Figure 2B). Fourteen studies [16, 19, 21, 23, 24, 27, 28, 30, 31, 33, 37, 39, 40, 45] comprising 10,410 CRC patients investigated the association between PLR and DFS. As shown in Figure 3, patients with high pretreatment PLR had significantly shorter DFS (pooled HR = 1.58, 95% CI: 1.31 – 1.92, *p*< 0.00001, I^2^=66%), suggesting that elevated PLR was associated with poor DFS.

### Impact of LMR on OS,CSS and DFS in CRC Patients

**Figure 4:**
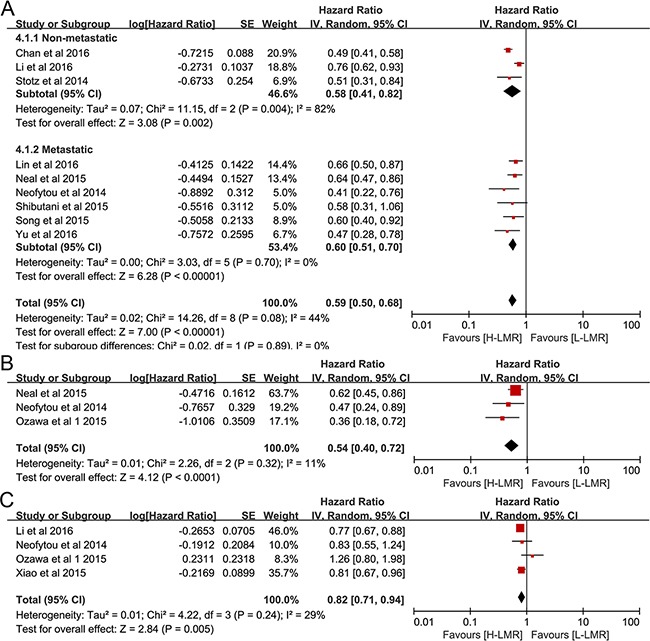
Forest plot reflects the association between LMR and OS **A**. CSS **B**. DFS **C**.

Nine studies [18, 20, 25, 26, 31, 35, 38, 39, 47] which included a total of 8667 CRC patients provided data for OS. As depicted in Figure 4, pooled data showed that elevated LMR was correlated with favorable OS in CRC patients(pooled HR = 0.59, 95% CI: 0.50 – 0.68, *p*< 0.00001, I^2^=44%, Figure 4A). Subgroup statistics indicated that this prognostic role of LMR was observed in both metastatic or non-metastatic CRC patients (pooled HR = 0.60, 95% CI = 0.51 – 0.70, *p*< 0.001 and pooled HR =0.58, 95% CI = 0.41 – 0.82, *p=*0.002, respectively, Figure 4A). The pooled statistics of three studies [36, 38, 39], which studied the correlation between LMR and CSS, suggested that elevated LMR was a prognostic factor for favorable CSS (pooled HR = 0.54, 95% CI: 0.40 – 0.72, *p*< 0.00001, I^2^=11%, Figure 4B). Our results also revealed that LMR was a predictor for prolonged DFS (pooled HR = 0.82, 95% CI: 0.71 – 0.94, *p*=0.005, I^2^=29%, Figure 4C).

### Subgroup analysis

Exploratory subgroup analyses were conducted according to geographic region (Asia and non-Asia), sample size (large and small), disease stage (metastatic/mixed and non-metastatic disease), methods for survival analysis(multivariable and univariate analysis), cut-off (≥185 and <185) and methods for determining cut-off (ROC/software analysis and referring to the previous study). However, results of the subgroup analysis for these variables did not alter the prognostic roles of PLR on OS and DFS and LMR on OS. While LMR was not associated with DFS in the non-Asian, small sample size, metastatic/mixed, univariate analysis and cut-off value≥3.0 subgroups. The difference is more likely clinically insignificant in these subgroups considering only four studies were used for this portion of the analyses. The details of the subgroup analyses are summarized in Table 2.

**Table 2:** Subgroup analyses for OS and DFS/RFS

		OS		I^2^	DFS/RFS		I^2^
		N	HR (95%CI, *P* value)		N	HR (95%CI, *P* value)	
PLR	Overall	20	1.57 (1.41-1.75, *p*<0.00001)	26%	14	1.58 (1.31-1.92, *p*<0.00001 )	66%
	Geographic region						
	Asia	12	1.60 (1.36-1.88, *p*<0.00001)	40%	9	1.50(1.19-1.90, *p*=0.0007)	68%
	Non-Asia	8	1.58 (1.39-1.80, *p*<0.00001)	0%	5	1.71 (1.24-2.35, *p*=0.001 )	58%
	Sample size						
	Large (n >200)	10	1.56 (1.31-1.86, *p*<0.00001 )	49%	9	1.66 (1.26-2.20, *p*=0.0004)	76%
	Small (n <200)	10	1.64 (1.44-1.87, *p*<0.00001)	0%	5	1.38 (1.14-1.68, *p*=0.0009)	5%
	Cut-off value						
	≥185*	12	1.66 (1.42-1.95, *p*<0.00001)	38%	5	1.93 (1.14-3.26, *p*=0.01)	87%
	<185	8	1.45 (1.26-1.66, *p*<0.00001)	0%	9	1.37 (1.19-1.56, *p*<0.00001)	0%
	Methods to determine cut-off						
	ROC/software analysis	8	1.53 (1.26-1.86, *p*<0.00001 )	54%	8	1.51 (1.19-1.91, *p*=0.0007)	68%
	RPS or NR	12	1.60 (1.41-1.81, *p*<0.00001)	0%	6	1.80 (1.20-2.69, *p*=0.005)	65%
	Disease stage						
	Non-metastatic	10	1.59 (1.32-1.91, *p*<0.00001)	45%	11	1.71 (1.29-2.25, *p*=0.0002)	73%
	Metastatic/mixed	10	1.54 (1.36-1.75, *p*<0.00001)	0%	3	1.34 (1.13-1.59, *p*=0.0007 )	0.06
	Variable type						
	Multivariable	16	1.58 (1.37-1.81, *p*<0.00001 )	38%	10	1.58 (1.26-1.98, *p*<0.00001)	73%
	Univariable	4	1.62 (1.39-1.89, *p*<0.00001)	0%	4	1.61 (1.18-2.18, *p*=0.002)	0%
LMR	Overall	9	0.59 (0.50-0.68, *p*<0.00001)	44%	4	0.82 (0.71-0.94, *p*=0.005)	29%
	Geographic region						
	Asia	6	0.66 (0.58-0.76, *p*<0.00001)	0%	3	0.83 (0.70-0.99, *p*=0.04)	52%
	Non-Asia	3	0.52 (0.42-0.64, *p*<0.00001)	32%	1	0.83 (0.55-1.24, *p*=0.36)	NA
	Sample size						
	Large (n >200)	5	0.61 (0.50-0.75, *p*<0.00001)	67%	2	0.78 (0.70-0.81, *p*<0.00001)	0%
	Small (n <200)	4	0.52 (0.40-0.68, *p*<0.00001)	0%	2	1.01 (0.67-1.52, *p*=0.97)	46%
	Cut-off value						
	≥3.00	5	0.58 (0.48-0.71, *p*<0.00001 )	0%	3	0.89 (0.70-1.13, *p*=0.33)	39%
	<3.00	4	0.61 (0.50-0.75, *p*<0.00001)	67%	1	0.77 (0.76-0.88, *p*=0.0002)	NA
	Disease stage						
	Non-metastatic	3	0.58 (0.41-0.82, *p*=0.002)	82%	2	0.78 (0.70-0.81, *p*<0.00001)	0%
	Metastatic/mixed	6	0.60 (0.51-0.70, *p*<0.00001)	0%	2	1.01 (0.67-1.52, *p*=0.97)	46%
	Variable type						
	Multivariable	8	0.58 (0.48-0.68, *p*<0.00001)	49%	3	0.83 (0.70-0.99, *p*=0.04)	52%
	Univariable	1	0.64 (0.47-0.86, *p*=0.003)	NA	1	0.83 (0.55-1.24, *p*=0.36)	NA

^*^median

### Sensitivity analysis

**Figure 5:**
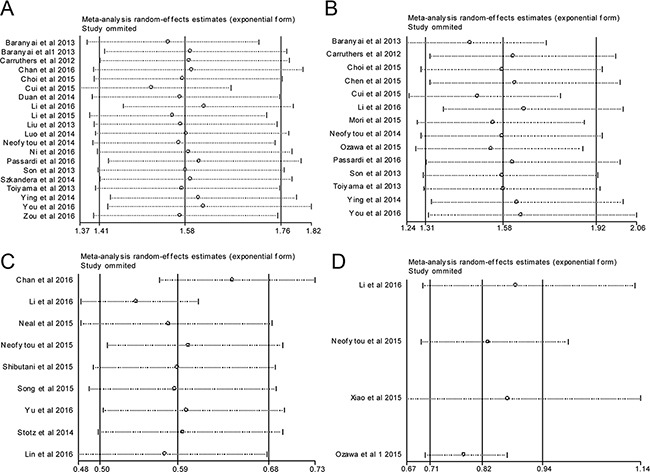
Sensitivity analysis for meta-analysis **A**. correlation of PLR with OS; **B**. correlation of PLR with DFS; **C**. correlation of LMR with OS; **D**. correlation of LMR with DFS.

Sensitivity analysis was performed by assessing the potential impact of individual studies on the pooled data. As illustrated in Figure 5, pooled HR was not significantly altered when each single study was withdrawn every time. Notably, there was substantial heterogeneity regarding the impact of LMR on DFS (I^2^=66%); however, exclusion of three studies [31, 37, 45] reduced the I^2^ to 0% and did not change the prognostic significance ( pooled HR =1.39, 95% CI=1.23–1.58, *p* <0.001).

### Publication bias

**Figure 6:**
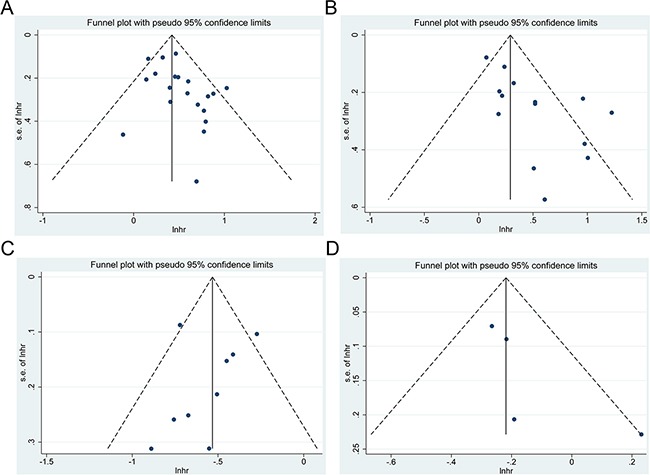
Funnel plot for publication bias **A**. correlation of PLR with OS; **B**. correlation of PLR with DFS; **C**. correlation of LMR with OS; **D**. correlation of LMR with DFS.

As shown in Figure 6, the funnel plots showed evidence for symmetry in studies of the impact of LMR on CRC survival, but not in studies of PLR, suggesting that a publication bias about for the effect of PLR on CRC outcomes may exist. Therefore, the Begg's and Egger's tests were conducted to assess the bias more precisely. Studies concerning PLR and pooled OS (Egger's test, *p*=0.048; Begg's test, *p*=0.127) and DFS (Egger's test, *p*=0.004; Begg's test, *p*=0.063) showed publication bias (Supplementary Table 1). After doing a trim fill analysis, we found that the pooled HR was 1.453 (95% CI = 1.286 −1.641, *p* <0.001) for OS and 1.206 (95% CI = 0.982 −1.482, *p*=0.074) for DFS, suggesting that a publication bias appeared to overestimate DFS.

## DISCUSSION

Recent studies [49–51] have shown correlation between the SIR and clinical outcomes in various cancers; However, conflicting evidence exists regarding the effects of PLR and LMR on the prognosis of CRC patients. In this meta-analysis of 33 studies which includes 15,404 cases, we reevaluated the prognostic roles of the PLR and LMR in CRC. The results of this study suggested that pretreatment PLR and LMR could be used as prognostic predictors in CRC patients. Elevated PLR was associated with poor OS and reduced DFS. On the contrary, high LMR was correlated with favorable OS, CSS and DFS. Analyses stratified by geographic region, sample size, different cut-off (≥185 and <185) and methods in determining cut-off did not alter the effects of PLR and LMR on OS and DFS.

Most of included studies (82%) were published in 2014 or later, highlighting the recent interest in investigating the prognostic values of PLR and LMR in CRC. To our knowledge, the meta-analysis is a more comprehensive update that systematically and quantitatively evaluates this topic. When assessing the impact of PLR on OS, the pooled HR of three studies which defined three risk categories (binary cut-offs) did not achieve statistical significance. This may be due to numerically lower HRs that apply per higher risk category compared with using a single cutoff [52]. We performed a sensitivity analysis, which indicated our results were robust. Publication bias was identified by a funnel plot and the Begg's and Egger's tests. The results revealed that studies concerning PLR and pooled OS and DFS showed publication bias, indicating that results, especially those regarding the impact of PLR on DFS, should be interpreted with caution.

The underlying mechanisms by which PLR and LMR influence the survival of CRC patients remains largely unknown. Several hypotheses have been put forward to explain the underlying biological basis. Thrombocytosis is commonly observed in cancer patients and is linked with decreased survival [53]. Platelets can release a myriad of growth factors which may facilitate cancer growth and dissemination. Orellana *et al*. [54] co-cultivated ovarian cancer cells with human platelets and found that platelet-cancer interactions contributed to the formation of metastatic foci. In addition, blockade of key platelet receptors attenuated ovarian cancer metastasis. Lymphocytopenia is a key component of a high PLR. Lymphocytes represent the cellular basis of cancer immunosurveillance. Compelling evidence indicates that lymphocytes induce cytotoxic cell death and inhibit tumor cell proliferation and migration, thereby dictating the host's immune response to cancer [55]. Decreased lymphocyte counts may lead to downregulation of the immune response against cancer. Monocytes may reflect the formation of tumor-associated macrophages(TAMs), which represent pivotal components of tumor microenvironment promoting progression [56]. Furthermore, PLR and LMR are representative indexes of SIR. Aberrant SIR is considered to be associated with cancer progression. In addition, systemic inflammation can decrease organ function in cancer patients; thus, poor oncologic outcomes are observed [57].

Several potential limitations of this study should be acknowledged. First, the major disadvantage of this study was the discordance of PLR and LMR cut-offs, which lead to inter-study heterogeneity. Second, patients receiving neoadjuvant chemotherapy were included in many of the studies, which may alter the course of the survival. Third, significant heterogeneity was found in publications studying the impact of PLR on OS and DFS. In addition, several disease conditions, including liver diseases or inflammatory diseases, may affect PLR and/or LMR. Some eligible studies did not control for these confounding factors.

## MATERIALS AND METHODS

### Literature search

Pubmed, Embase, and CNKI were systematically searched for literature up to June 2016. The main medical subject heading (Mesh) terms and text words included colorectal cancer, lymphocyte, platelets, monocytes and prognosis. The search strategies were summarized in Supplementary Appendix. The languages of articles were limited to English and Chinese. The bibliographies of relevant articles were also searched manually for additional eligible studies. Inter-reviewer agreement was evaluated using Cohen's kappa. Any disagreements were discussed and arbitrated by a second reviewer.

### Study selection

A study was considered eligible only if the publication met all of the following criteria: (a) patients were pathologically diagnosed with CRC; (b) pretreatment PLR and/or LMR and cutoff values were reported; (c) PLR and/or LMR were used as prognostic indicators of OS, CSS or DFS; (c) hazard ratios and 95% confidence intervals were reported in text. The exclusion criteria were as follows: (a) PLR and/or LMR were reported as continuous variables; (b) studies had overlapping or duplicated data; (c) non-research articles or studies that were based on animal or human cell lines; (d) publications were not subjected to peer-review (dissertations or theses).

### Data extraction

Two investigators independently gathered data. The following data were extracted: publication details (first author's surname, year of publication, geographic region of study), population characteristics (patients number, age, and sex), cancer and follow-up data (cancer site, stage, treatment strategy, median/mean follow-up duration, survival analysis), PLR and/or LMR data (assessment method and cut-off values), cut-off values were used to determine ‘high’ versus ‘low’ PLR and LMR.

### Qualitative assessment

The quality of each of the included studies was assessed using the Newcastle–Ottawa Quality Assessment Scale (NOS, Supplementary Table 2) [58], which includes 3 criteria, namely, selection (0–4 points), comparability (0–2 points) and outcomes (0–3 points). NOS scores≧6 were defined as high-quality. (Supplementary Table 3).

### Statistical analysis

The HR with 95% CI was directly retrieved from each of the article. Pooled HR was calculated using the generic inverse variance and random-effect model. A combined HR >1 implied a worse prognosis in the group with elevated PLR or LMR. Inter-study heterogeneity was measured by performing the c2-based Cochran's Q test and Higgins’ *I^2^* statistics. A *P*-value <0.10 and/or *I^2^*>50% indicated significant heterogeneity. Publication bias was assessed with visual inspection of funnel plots and precisely evaluated by Egger's and Begg's tests. A *P*-value < 0.05 in the Z test for pooled HR, or no overlap of the 95% CI with 1 was considered statistically significant. This study adhered to the PRISMA guidelines and all data analysis was performed using Review Manager 5.2 (Cochrane Collaboration, London, UK) and Stata 12.0 software (Stata Corporation, College Station, TX, USA).

## CONCLUSIONS

In summary, pretreatment PLR and LMR could be used as prognostic predictors in CRC patients. Elevated PLR was associated with poor OS and DFS. In contrast, high LMR correlated with favorable OS, CSS and DFS. Further studies are necessary to confirm these findings and elucidate the underlying biology.

## SUPPLEMENTARY MATERIALS TABLES




