# Supplementary material for: The clinical use of the platelet/lymphocyte ratio and lymphocyte/monocyte ratio as prognostic predictors in colorectal cancer: a meta-analysis
Source: Oncotarget. 2017 Feb 14;8(12):20011–24. doi: 10.18632/oncotarget.15311 (PMC5386740; doi:10.18632/oncotarget.15311)
Supplement: Supplementary file 1 [file oncotarget-08-20011-s001.pdf]

The clinical use of the platelet/lymphocyte ratio and lymphocyte/monocyte ratio as prognostic predictors in colorectal cancer: a meta-analysis

SUPPLEMENTARY APPENDIX

2016/6/23

Pubmed

#1: Search (((((((((((((((("Colorectal Neoplasms"[Mesh]) OR Colorectal Neoplasms[Title/Abstract]) OR Neoplasms, Colorectal[Title/Abstract]) OR Colorectal Neoplasm[Title/Abstract]) OR Neoplasm, Colorectal[Title/Abstract]) OR Colorectal Tumors[Title/Abstract]) OR Colorectal Tumor[Title/Abstract]) OR Tumor, Colorectal[Title/Abstract]) OR Tumors, Colorectal[Title/Abstract]) OR Colorectal Carcinoma[Title/Abstract]) OR Carcinoma, Colorectal[Title/Abstract]) OR Carcinomas, Colorectal[Title/Abstract]) OR Colorectal Carcinomas[Title/Abstract]) OR Colorectal Cancer[Title/Abstract]) OR Cancer, Colorectal[Title/Abstract]) OR Cancers, Colorectal[Title/Abstract]) OR Colorectal Cancers[Title/Abstract] 189140

#2: Search ((((((((((((((((("Colonic Neoplasms"[Mesh]) OR Colonic Neoplasms[Title/Abstract]) OR Colonic Neoplasm[Title/Abstract]) OR Neoplasm, Colonic[Title/Abstract]) OR Neoplasms, Colonic[Title/Abstract]) OR Colon Neoplasms[Title/Abstract]) OR Colon Neoplasm[Title/Abstract]) OR Neoplasm, Colon[Title/Abstract]) OR Neoplasms, Colon[Title/Abstract]) OR Cancer of Colon[Title/Abstract]) OR Colon Cancers[Title/Abstract]) OR Cancer of the Colon[Title/Abstract]) OR Colonic Cancer[Title/Abstract]) OR Cancer, Colonic[Title/Abstract]) OR Cancers, Colonic[Title/Abstract]) OR Colonic Cancers[Title/Abstract]) OR Colon Cancer[Title/Abstract]) OR Cancer, Colon[Title/Abstract]) OR Cancers, Colon[Title/Abstract]

111378

#3: Search (((((((((((((((((((("Rectal Neoplasms"[Mesh]) OR Rectal Neoplasms[Title/Abstract]) OR Neoplasm, Rectal[Title/Abstract]) OR Rectal Neoplasm[Title/Abstract]) OR Rectum Neoplasms[Title/Abstract]) OR Neoplasm, Rectum[Title/Abstract]) OR Neoplasms, Rectum[Title/Abstract]) OR Rectum Neoplasm[Title/Abstract]) OR Neoplasms, Rectal[Title/Abstract]) OR Rectal Tumors[Title/Abstract]) OR Rectal Tumor[Title/Abstract]) OR Tumor, Rectal[Title/Abstract]) OR Tumors, Rectal[Title/Abstract]) OR Cancer of Rectum[Title/Abstract]) OR Rectum Cancers[Title/Abstract]) OR Rectal Cancer[Title/Abstract]) OR Cancer, Rectal[Title/Abstract]) OR Cancers, Rectal[Title/Abstract]) OR Rectal Cancers[Title/Abstract]) OR Rectum Cancer[Title/Abstract]) OR Cancer, Rectum[Title/Abstract]) OR Cancers, Rectum[Title/Abstract]) OR Cancer of the Rectum[Title/Abstract] 63139

#4: Search ((((((((Blood Platelets[MeSH Terms]) OR Blood Platelets[Title/Abstract]) OR Blood Platelet[Title/Abstract]) OR Platelet, Blood[Title/Abstract]) OR Platelets, Blood[Title/Abstract]) OR Thrombocytes[Title/Abstract]) OR Thrombocyte[Title/Abstract]) OR Platelets[Title/Abstract]) OR Platelet[Title/Abstract]

198359

#5: Search ((((((Lymphocytes[MeSH Terms]) OR Lymphocytes[Title/Abstract]) OR Lymphocyte[Title/Abstract]) OR Lymphoid Cells[Title/Abstract]) OR Cell, Lymphoid[Title/Abstract]) OR Cells, Lymphoid[Title/Abstract]) OR Lymphoid Cell[Title/Abstract]

611835

#6: Search ((monocyte[MeSH Terms]) OR monocyte[Title/Abstract]) OR monocyte[Title/Abstract] 86094

#7: Search (((((((((((((("Prognosis"[Mesh]) OR Prognosis[Title/Abstract]) OR Prognoses[Title/Abstract]) OR Prognostic[Title/Abstract]) OR Outcome[Title/Abstract]) OR Survival[Title/Abstract]) OR Overall survival[Title/Abstract]) OR OS[Title/Abstract]) OR Cancer-specific survival[Title/Abstract]) OR CSS[Title/Abstract]) OR Progression-free survival[Title/Abstract]) OR PFS[Title/Abstract]) OR Disease-free survival[Title/Abstract]) OR DFS[Title/Abstract]) OR Mortality[Title/Abstract]) OR Recurrence[Title/Abstract] 2768624

#8:#1 or #2 or #3 227557

#9:#4 and #5 9289

#10:#5 and #6 24322

#11:#9 OR #10 32509

#12:#7 AND #8 AND #11 106

EMBASE

#1 Search: 'colorectal cancer'/exp OR 'colorectal cancer' OR 'colorectal neoplasms':ab,ti OR 'neoplasms, colorectal':ab,ti OR 'colorectal neoplasm':ab,ti OR 'neoplasm, colorectal':ab,ti OR 'colorectal tumors':ab,ti OR 'colorectal tumor':ab,ti OR 'tumor, colorectal':ab,ti OR 'tumors, colorectal':ab,ti OR 'colorectal carcinoma':ab,ti OR 'carcinoma, colorectal':ab,ti OR 'carcinomas,

colorectal':ab,ti OR 'colorectal carcinomas':ab,ti OR 'colorectal cancer':ab,ti OR 'cancer, colorectal':ab,ti OR 'cancers, colorectal':ab,ti OR 'colorectal cancers':ab,ti 149817

#2 Search: 'colon cancer'/exp OR 'colon cancer' OR 'colonic neoplasms':ab,ti OR 'colonic neoplasm':ab,ti OR 'neoplasm, colonic':ab,ti OR 'neoplasms, colonic':ab,ti OR 'colon neoplasms':ab,ti OR 'colon neoplasm':ab,ti OR 'neoplasm, colon':ab,ti OR 'neoplasms, colon':ab,ti OR 'cancer of colon':ab,ti OR 'colon cancers':ab,ti OR 'cancer of the colon':ab,ti OR 'colonic cancer':ab,ti OR 'cancer, colonic':ab,ti OR 'cancers, colonic':ab,ti OR 'colonic cancers':ab,ti OR 'colon cancer':ab,ti OR 'cancer, colon':ab,ti OR 'cancers, colon':ab,ti 212626

#3 Search: 'rectum cancer'/exp OR 'rectum cancer' OR 'rectal neoplasms':ab,ti OR 'neoplasm, rectal':ab,ti OR 'rectal neoplasm':ab,ti OR 'rectum neoplasms':ab,ti OR 'neoplasm, rectum':ab,ti OR 'rectum neoplasm':ab,ti OR 'neoplasms, rectal':ab,ti OR 'rectal tumors':ab,ti OR 'rectal tumor':ab,ti OR 'tumor, rectal':ab,ti OR 'tumors, rectal':ab,ti OR 'cancer of rectum':ab,ti OR 'rectum cancers':ab,ti OR 'rectal cancer':ab,ti OR 'cancer, rectal':ab,ti OR 'cancers, rectal':ab,ti OR 'rectal cancers':ab,ti OR 'rectum cancer':ab,ti OR 'cancer, rectum':ab,ti OR 'cancers, rectum':ab,ti OR 'cancer of the rectum':ab,ti 160832

#4 Search: 'blood platelets'/exp OR 'blood platelets':ab,ti OR 'blood platelet':ab,ti OR 'platelet, blood':ab,ti OR 'platelets, blood':ab,ti OR thrombocytes:ab,ti OR thrombocyte:ab,ti OR platelets:ab,ti

145501

#5 Search: 'lymphocytes'/exp OR 'lymphocytes':ab,ti OR 'lymphocyte':ab,ti OR 'lymphoid cell':ab,ti OR 'cell, lymphoid':ab,ti OR 'cells, lymphoid':ab,ti OR 'lymphoid cells':ab,ti

802339

#6 Search: 'monocyte'/exp OR monocytes:ab,ti OR monocyte:ab,ti 134166

#7 Search: 'prognosis'/exp OR prognosis:ab,ti OR prognoses:ab,ti OR prognostic:ab,ti OR outcome:ab,ti OR survival:ab,ti OR 'overall survival':ab,ti OR os OR 'cancer-specific survival':ab,ti OR css:ab,ti OR 'progression-free survival':ab,ti OR pfs:ab,ti OR 'disease-free survival':ab,ti OR dfs:ab,ti OR mortality:ab,ti OR recurrence:ab,ti

2987347

#8 Search:#1 OR #2 OR #3 261589

#9 Search:#4 AND #5 9694

#10 Search:#5 AND #6 48335

#11 Search:#9 OR #10 55745

#12 Search:#7 AND #8 AND #11 176

CNKI 64

SUPPLEMENTARY TABLES

Supplementary Table 1: Publication bias for outcomes was determined by the Begg and Egger tests

Variable	Outcomes	Begg's test	Egger's test		
		P value	t-value	95%CI	*P* value
PLR	OS	0.127	2.11	-0.11-2.11	0.048
	DFS/RFS	0.063	3.35	0.89-3.72	0.004
LMR	OS	0.118	-0.44	-3.11-2.13	0.672
	DFS/RFS	0.089	1.89	-2.44-6.27	0.199

Supplementary Table 2: Newcastle – Ottawa quality assessment scale

Item
**Selection**
(1) Representativeness of the exposed cohort (a) Truly representative of the average 'colorectal can patients ' in the community (1 star) (b) Somewhat representative of the average 'colorectal can patients ' in the community (1 star) (c) Selected group of users (e.g. nurses, volunteers) (d) No description of the derivation of the cohort
(2) Selection of the non-exposed cohort (a) Drawn from the same community as the exposed cohort (1 star) (b) Drawn from a different source (c) No description of the derivation of the non-exposed cohort
(3) Ascertainment of exposure () (a) Secure record (eg. surgical records) (1 star) (b) Structured interview (1 star) (c) Written self-report (d) No description
(4) Demonstration that outcome of interest was not present at start of study (a) Yes (1 star) (b) No
**Comparability**
(1) Comparability of cohorts on the basis of the design or analysis (a) Study controls for 'metastasis or recurrence' (1 star) (b) Study controls for any additional factor (1 star) (inflammatory diseases, stage, etc.)
**Outcome**
(1) Assessment of outcome (Death or recurrence) (a) Independent blind assessment (1 star) (b) Record linkage (1 star) (c) Self-report (d) No description
(2) Was follow-up long enough for outcomes to occur? (a) Yes ('3 years') (1 star) (b) No
(3) Adequacy of follow-up of cohorts (a) Complete follow-up – all subjects accounted for (1 star) (b) Subjects lost to follow-up unlikely to introduce bias – small number lost '(25%)' or description provided of those lost (1 star) (c) Follow-up rate '<75%' and no description of those lost (d) No statement

Notes: *a study can be awarded a maximum of one point for each numbered item within the "selection" and "Outcome" categories, and a maximum of two points can be given for "comparability".

Supplementary Table 3: Methodological quality of all studies based on the Newcastle-Ottawa scale for assessing the quality of cohort trials

See Supplementary File 1
