# Supplementary material for: The clinical use of the platelet/lymphocyte ratio and lymphocyte/monocyte ratio as prognostic predictors in colorectal cancer: a meta-analysis
Source: Oncotarget. 2017 Feb 14;8(12):20011–24. doi: 10.18632/oncotarget.15311 (PMC5386740; doi:10.18632/oncotarget.15311)
Supplement: Supplementary file 2 [file oncotarget-08-20011-s002.docx]

**Supplemental table 3. Methodological quality of all studies based on the Newcastle-Ottawa scale for assessing the quality of cohort trials**

| **Author, year** | **Representativeness of exposed cohort** | **Selection of non-exposed cohort** | **Assessment of exposure** | **Outcome not present at start of study** | **Comparability based on the design or analysis** | **Assessment of outcome** | **Follow-up long enough for outcomes** | **Adequacy of follow-up** | **Total**  **score** |
| --- | --- | --- | --- | --- | --- | --- | --- | --- | --- |
| Baranyai *et al.,* 2103 | 1 | 1 | 1 | 1 | 1 | 1 | 0 | 0 | 6 |
| Carruthers *et al.,* 2012 | 1 | 1 | 1 | 1 | 0 | 1 | 1 | 0 | 6 |
| Chan *et al.,* 2016 | 1 | 1 | 1 | 1 | 1 | 1 | 1 | 0 | 7 |
| Choi *et al.*, 2015 | 1 | 1 | 1 | 1 | 2 | 1 | 1 | 0 | 8 |
| Chen *et al.*, 2015 | 1 | 1 | 1 | 1 | 2 | 0 | 0 | 0 | 6 |
| Cui *et al.*, 2015 | 1 | 1 | 1 | 1 | 2 | 1 | 0 | 0 | 7 |
| Duan *et al.*, 2014 | 1 | 1 | 1 | 1 | 0 | 0 | 1 | 0 | 5 |
| Kwon *et al.*, 2012 | 1 | 1 | 1 | 1 | 2 | 1 | 0 | 1 | 8 |
| Li *et al.*, 2016 | 1 | 1 | 1 | 1 | 1 | 1 | 1 | 1 | 8 |
| Li *et al.*, 2015 | 1 | 1 | 1 | 1 | 2 | 1 | 0 | 0 | 7 |
| Liu *et al.*, 2015 | 1 | 1 | 1 | 1 | 1 | 1 | 0 | 0 | 6 |
| Lin *et al.*, 2016 | 1 | 1 | 1 | 1 | 2 | 1 | 1 | 1 | 9 |
| Luo *et al.*, 2014 | 1 | 1 | 1 | 1 | 0 | 1 | 0 | 0 | 6 |
| Mori *et al.*, 2015 | 1 | 1 | 1 | 1 | 2 | 1 | 0 | 0 | 7 |
| Neal *et al.*, 2015 | 1 | 1 | 1 | 1 | 2 | 1 | 0 | 1 | 8 |
| Neofytou *et al.*, 2014 | 1 | 1 | 1 | 1 | 2 | 1 | 1 | 1 | 9 |
| Neofytou *et al.*, 2015 | 1 | 1 | 1 | 1 | 2 | 1 | 1 | 1 | 9 |
| Ni *et al.*, 2016 | 1 | 1 | 1 | 1 | 2 | 1 | 1 | 0 | 8 |
| Ozawa *et al.*, 2015 | 1 | 1 | 1 | 1 | 2 | 1 | 1 | 1 | 9 |
| Ozawa *et al.*, 1 2015 | 1 | 1 | 1 | 1 | 2 | 1 | 1 | 1 | 9 |
| Passardi *et al.*, 2016 | 1 | 1 | 1 | 1 | 1 | 1 | 1 | 1 | 8 |
| Shibutani *et al.*, 2015 | 1 | 1 | 1 | 1 | 1 | 1 | 0 | 0 | 6 |
| Son *et al.*, 2013 | 1 | 1 | 1 | 1 | 1 | 1 | 1 | 0 | 7 |
| Song *et al.*, 2015 | 1 | 1 | 1 | 1 | 1 | 1 | 1 | 0 | 7 |
| Stotz *et al.*, 2014 | 1 | 1 | 1 | 1 | 1 | 1 | 1 | 1 | 8 |
| Sun *et al.*, 2014 | 1 | 1 | 1 | 1 | 1 | 0 | 1 | 1 | 7 |
| Szkandera *et al.*, 2014 | 1 | 1 | 1 | 1 | 1 | 1 | 1 | 1 | 8 |
| Toiyama *et al.*, 2013 | 1 | 1 | 1 | 1 | 1 | 0 | 1 | 1 | 7 |
| Xiao *et al.*, 2015 | 1 | 1 | 1 | 1 | 1 | 1 | 1 | 0 | 7 |
| Ying *et al.*, 2014 | 1 | 1 | 1 | 1 | 2 | 1 | 0 | 0 | 7 |
| You *et al.*, 2016 | 1 | 1 | 1 | 1 | 2 | 1 | 1 | 0 | 8 |
| Yu *et al.*, 2016 | 1 | 1 | 1 | 1 | 1 | 1 | 0 | 0 | 6 |
| Zou *et al.*, 2016 | 1 | 1 | 1 | 1 | 2 | 1 | 1 | 0 | 8 |
